# Pelvic Girdle Pain, Hypermobility Spectrum Disorder and Hypermobility-Type Ehlers-Danlos Syndrome: A Narrative Literature Review

**DOI:** 10.3390/jcm9123992

**Published:** 2020-12-09

**Authors:** Ahmed Ali, Paul Andrzejowski, Nikolaos K. Kanakaris, Peter V. Giannoudis

**Affiliations:** 1 Academic Department of Trauma and Orthopaedics, School of Medicine, University of Leeds, Floor D, Clarendon Wing, Leeds General Infirmary, Great George Street, Leeds LS1 3EX, UK; paul.andrzejowski@nhs.net (P.A.); n.kanakaris@nhs.net (N.K.K.); 2 NIHR Leeds Biomedical Research Unit, Chapel Allerton Hospital, Leeds LS7 4SA, UK

**Keywords:** pelvic girdle pain, pelvic pain, pelvic instability, pubic symphysis, sacroiliac joint, fusion, hypermobility, Ehlers-Danlos Syndrome

## Abstract

Pelvic girdle pain (PGP) refers specifically to musculoskeletal pain localised to the pelvic ring and can be present at its anterior and/or posterior aspects. Causes such as trauma, infection and pregnancy have been well-established, while patients with hypermobile joints are at greater risk of developing PGP. Research exploring this association is limited and of varying quality. In the present study we report on the incidence, pathophysiology, diagnostic and treatment modalities for PGP in patients suffering from Hypermobility Spectrum Disorder (HSD) and Hypermobility-Type Ehlers-Danlos Syndrome (hEDS). Recommendations are made for clinical practice by elaborating on screening, diagnosis and management of such patients to provide a holistic approach to their care. It appears that this cohort of patients are at greater risk particularly of mental health issues. Moreover over, they may require a multidisciplinary approach for their management. Ongoing research is still required to expand our understanding of the relationship between PGP, HSD and hEDS by appropriately diagnosing patients using the latest updated terminologies and by conducting randomised control trials to compare outcomes of interventions using standardised patient reported outcome measures.

## 1. Introduction

Pelvic girdle pain (PGP) refers specifically to musculoskeletal pain localised to the level of the posterior iliac crest and the gluteal fold, and occasionally over the anterior and posterior elements of the bony pelvis [1]. There are many causes of pelvic pain overall, including trauma, infections of soft tissue, bone or underlying viscera, lower-back pain syndromes, pregnancy, joint hypermobility and disorders of connective tissues [1,2]. Given the broad range of possible causes, when managing these patients, it is important for an extensive history and clinical examination to be conducted, followed by appropriate investigations to facilitate accurate diagnosis—and to distinguish between aetiologies.

**Figure 1:**
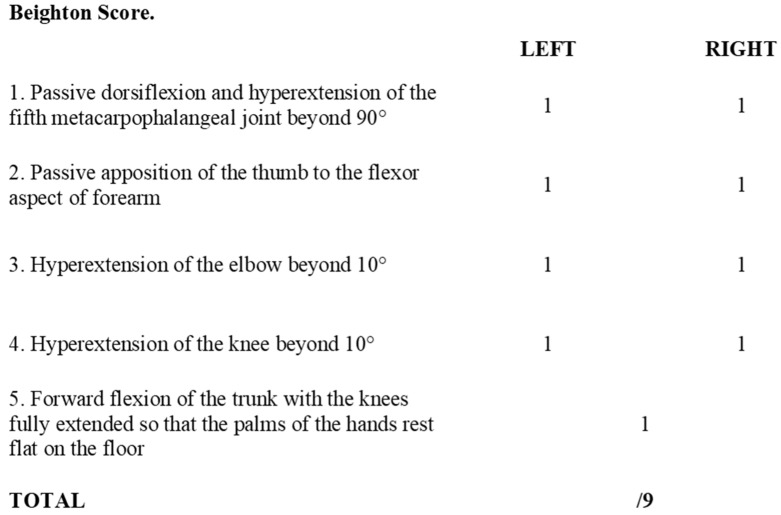
Beighton score. Adapted from Beighton et al., 1973 [14].

Joint hypermobility (JH) places a great level of strain on an affected individual’s musculoskeletal system. JH occurs secondary to increased elasticity of the soft tissues that support a joint, tendons and ligaments, which allows a greater range of motion [3]. A tool such as the Beighton score which uses five simple manoeuvres can be used to screen individuals for hypermobile joints in clinical practice (Figure 1). Complications can include joint pain, dislocations and instability which can limit daily activities and quality of life. Previous research has highlighted that there may be a link between JH and development of osteoarthritis secondary to the increased stress placed on joints, however studies have reported mixed findings [4,5,6,7,8,9]. Although JH has disadvantages linked to increased risk of injury and anxiety, particularly in contact sports, in others such as gymnastics and ballet it may in fact be advantageous [10,11,12,13].

Patients with joint hypermobility (JH) are classified according to their position on the hypermobility spectrum, which ranges in severity from asymptomatic joint hypermobility, to hypermobility-type Ehlers-Danlos Syndrome (hEDS) [15]. This allows for inclusion of patients with a less severe constellation of symptoms, who may not necessarily meet criteria for diagnosis of a specific connective tissue disorder (Table 1). 

The classification framework for overall Joint Hypermobility (JH) and Ehlers-Danlos Syndrome subtypes were updated in 2017, before which the definitions were quite different. Prior to this, the commonly used terms were ‘joint hypermobility syndrome’ and hEDS alone. [15,16] One must bear this in mind when reading older literature.

Ehlers-Danlos Syndrome (EDS) is a genetically inherited connective tissue disorder characterised by hypermobile joints, friable tissues and hyperextensible skin. The first attempt to classify the condition was in 1936 by Weber, who termed it ‘Ehlers-Danlos Syndrome’ in honour of early published cases by these authors [17] In 2017, the current revised classification known as the international classification of EDS was created [16], which updated the previous set of definitions known as the 1998 Villefranche Nosology. This builds on an increased understanding of EDS: further subclassifications were generated, which refer to inheritance patterns, genetic abnormalities and affected proteins. The 2017 international classification lists clinical criteria that are suggestive of 13 EDS subtypes. The five most common subtypes are summarised in Table 2. The revised 2017 classification has clear, defined criteria for the diagnosis of hEDS, and a less-restrictive diagnosis criteria for HSD when patients may present with some features of hEDS, but not enough to be given a diagnosis of hEDS [15,16]. It is important to note that joint hypermobility (JH) can present in several subtypes of EDS, and not just hEDS [15,16].

As there is no investigation to diagnose hEDS, it is still diagnosed clinically. Attempts to find a genetic test have proved difficult due to the clinical and genetic heterogeneity of this syndrome [18,19,20]. An in vitro study utilising skin biopsies from patients with hEDS and hypermobility spectrum disorder (HSD) has identified a possible signalling pathway (αvβ3-ILK-Snail1/Slug axis) which promotes the transition of fibroblasts to myofibroblasts in hEDS and HSD patients, which was absent in biopsies from patients with classical EDS and vascular EDS [21]. Although further research is required on a larger sample of patients, this provides some insight into future directions of molecular testing which could support a clinical diagnosis of hEDS and HSD. Interestingly, further research is still required to differentiate between hEDS and HSD.

Given the relatively recent changes in 2017 used to describe JH, HSD and hEDS, it is difficult to determine the exact prevalence of these conditions. The distribution of hypermobility between the genders is however well reported to show a female predominance [14,22,23,24,25,26,27]. This greater prevalence in females has identified an increased concern related to pregnancy. Studies have found that females with JH are at increased odds of developing PGP during pregnancy than those who do not have JH [28]. A study investigating pregnancy complications in those with EDS identified that the prevalence of pelvic pain and instability was almost four-times greater than those without EDS [29]. Small case series have further data to support this with up to 88% of hEDS patients experiencing PGP during pregnancy [30]. The current available data is limited by study design and would benefit from larger prospective studies using the newer 2017 EDS criteria for diagnosis. This may however prove challenging due to undiagnosed cases, limited understanding, and rarity of cases.

The purpose of our narrative literature review is to explore the current incidence, pathophysiology, diagnostic and treatment modalities available for musculoskeletal pelvic girdle pain and HSD/hEDS, identify potential relationships between them, and finally to discuss gaps for future research.

## 2. Literature Search Method

A literature search was conducted on the 9 September 2020, using MEDLINE and EMBASE databases. The dates for this search were from 1 January 1970 to 9 September 2020. Given the variety of names used for pelvic girdle pain, a broad selection was used to identify as many relatable papers as possible. These were attached to the common keyword ‘hypermobility’ to identify papers which discuss or investigate hEDS or any other sub-category of the HSD. The following Boolean search was conducted: ((pelvic girdle pain) OR (pelvic pain) OR (pelvic instability) OR (pubis symphysis dysfunction) OR (pubic symphysis dysfunction) OR (sacroiliac dysfunction)) AND (hypermobility). This translates into the following MeSH terms used: Pelvic Girdle Pain (entry term Symphysis Pubis Dysfunction), Joint Instability (entry term Hypermobility). This was supplemented by a manual search of relevant citations from papers identified via MEDLINE/EMBASE, and focused searching using Google Scholar in order to identify the most current guidelines and evidence used in the diagnosis and management of PGP, HSD and hEDS.

Studies were excluded due to lack of access to full articles, language not being English, or them being deemed irrelevant after closer investigation of the full articles. Due to the nature of the broad search and the overlap of terminology used in past literature, many of the excluded articles included those discussing non-musculoskeletal causes of pelvic pain, such as gynaecological, urological and gastrointestinal for example. Extracted details of interest included studies reporting on prevalence of hypermobility in musculoskeletal pelvic girdle pain populations, as well as papers elaborating on the pathophysiology of this association.

## 3. Results

Overall, 112 results were generated from this search. After screening relevance and eligibility from the titles and abstracts for these articles, 23 were identified for further investigation. Of these 23 articles, 10 were deemed eligible and related to our literature review, which are summarised in Table 3 [28,31,32,33,34,35,36,37,38].

### 3.1. Epidemiology

There are no current studies quantifying the accurate prevalence specifically of hEDS and HSD within the general population. This could be a difficult task for several reasons including recent changes to definitions of hEDS and HSD, undiagnosed cases, reliability of diagnosis, limited awareness of the disease, and coding issues where patients are labelled with EDS and not the particular subtype. A study by Demmler et al. (2015) did attempt, though, to investigate the diagnosed prevalence of EDS and HSD in Wales, UK [24]. The findings of this study should however be interpreted with caution. This paper said that the diagnosis of ‘joint hypermobility syndrome’ (JHS) was common, and that the combined prevalence of *all* types of EDS was rare. The paper itself confuses the diagnosis of JHS with HSD and hEDS, and also states that HSD/EDS is common, and was challenged for inaccurate statements: combining a common condition with a rare one and stating that together they are common does not make sense. Furthermore, given the change in the 2017 criteria definitions, it is impossible to define how HSD and hEDS split out from JHS. It is estimated that the combined prevalence of all EDS subtypes is 1 in 5000, of which an estimated 80–90% are believed to be hEDS [41,42].

Epidemiological studies have estimated JH to have a prevalence between 2–35% [22,43,44,45,46]. Studies investigating pelvic pain in females have suggested that generalised-HSD can affect up to 24% of women, which may imply this to be an insignificant factor if not significant from general population figures [39]. This study did not contain an asymptomatic control population to compare prevalence of joint hypermobility.

PGP is a common feature experienced by pregnant women, with reports of it affecting between 23–65% of pregnant ladies [47,48]. The prevalence of PGP during pregnancy is found to be higher in individuals suffering from HSD (26%) compared to those who were controls (7%) [29]. Although PGP resolves for many women postpartum, in a select few it can persist. There have been reports of 8.6% of pregnant women experiencing PGP 2-years postpartum, and in other populations 10% experiencing PGP 11 years after birth [49,50]. In the latter study population, 29% of women with continuous PGP were known to have a diagnosis of JH.

The exact incidence and prevalence of HSD/hEDS has been difficult to establish due to the different definitions and diagnostic means used. The diagnosis has largely become one of exclusion once visceral, musculoskeletal, soft tissue infection and obstetric complications are investigated before HSD/hEDS is labelled as one’s cause of pelvic girdle pain.

### 3.2. Aetiology and Pathophysiology

#### 3.2.1. Hypermobility-Type Ehlers-Danlos Syndrome and Hypermobility Spectrum Disorder

EDS is a group of inherited connective tissue disorders affecting proteins in the extracellular matrix, including collagen. Given the wide variety of proteins that can be affected, this disease can present with several phenotypes. This has led to many subclassifications of this condition such as classical, vascular and hypermobile variants. The 2017 International Classification System for EDS aims to not only identify each subtype on clinical presentation, but also genetic and molecular testing for diagnostic confirmation of each subtype. The most common inheritance pattern of transmission is autosomal dominant, as is the case for hEDS, which is the focus of this review paper. Autosomal recessive and de novo mutations have also been reported [16]. Unlike other EDS subtypes, no associated genetic mutations have currently been identified for hEDS. Haploinsufficiency and missense variants of tenascin-X, which is an extracellular matrix protein encoded by the TNXB gene, has been reported in a handful of hEDS phenotype individuals [51,52]. However, variations in penetrance were seen and tenascin-X deficiency accounts for a minority of hEDS cases without a clear understanding of it physiologic process. Variations of the LZTS1 gene have also been identified in a family suffering hEDS; however the process again is not completely understood [53]. The diagnosis of hEDS remains clinical with strict criteria from the 2017 international classification system [16].

The 2017 international classification system for EDS also provides advice for the diagnosis of HSD as suggested by the paper ‘*A framework for the classification of joint hypermobility and related conditions*’ by Castori et al. which was published in the same issue as the EDS classification system [15]. This framework elaborates on JH classification as asymptomatic, part of well-defined syndromes and symptomatic JH which does not meet any criterion for a syndrome. The latter group is where the diagnosis of HSD is suggested. HSD itself has several subgroups being a spectrum, depending on the pattern and locations of hypermobile joints. It has been identified that the composition, proportion, structure and distribution of different collagen types are altered in hypermobile individuals, with an increased ratio of type III collagen to type I collagen [54]. The phenotype of JH is however influenced by multiple factors such as age, weight and training, and twin studies by Hakim et al. suggest strong genetic traits but with multifactorial influences resulting in concordance rates of 60% and 36% in monozygotic and dizygotic twins respectively [42,55].

#### 3.2.2. Pelvic Girdle Pain

Studies investigating the pelvic joints and hypermobility have found that pelvic pain is often secondary to altered biomechanics and transmission of forces via the spine [36]. This is thought to be a result of asymmetry in the pelvic ligaments, the extent of which was found to correlate with the pain score reported [56,57]. Radiographical studies investigating the physiological motion of the pubic symphysis have reported that asymptomatic individuals can have up to five-millimetres of motion at the pubic symphysis [58]. This study highlights the increased risk of patients with JH suffering from pelvic pain, due to unilateral or bilateral disruption of sacroiliac joint (SIJ), and/or the pubic symphysis secondary to increased range of motion beyond normal limits.

Theories have suggested that altered biomechanical loading and kinematics secondary to JH and instability can overload and injure joints, as repetitive microtrauma to its supporting structures can cause soft tissue injuries and arthralgias [59]. This is a form of overuse injury secondary to the increased flexibility, which can be linked to altered lumbopelvic movements in a hypermobile SIJ [36]. Impaired proprioception has also been found in patients with JH, and it has been identified as a risk factor for injury [60,61,62].

Soft tissue structures surrounding the pelvis play a primary role in its stabilisation. They can be divided into subgroups known as the longitudinal, anterior oblique and posterior oblique pelvic slings which group muscles to their particular role in stabilisation [36]. Poor coordination, or differences in their strength can lead to pelvis instability by impairing the tension on ligaments and tendons. In hypermobile patients, the laxity of soft tissues impairs the compressive forces normally generated to stabilise the pelvis. This results in altered transfer of axial load from the spine to the lower limbs via the SIJ, making the joint prone to degenerative disease [36].

The exact mechanisms that lead to the development of PGP from pregnancy remain uncertain. A variety of approaches have been proposed that suggest hormonal, biomechanical, traumatic, metabolic, genetic, and degenerative etiologic implications [63]. The hormone Relaxin is thought to be significant due to its effects on collagen remodelling, resulting in increased elasticity of soft tissues to facilitate delivery. Studies that have investigated the role of Relaxin and its relation to PGP, however, have determined that it is unlikely to be a cause for musculoskeletal disease, and that levels of Relaxin are not predictors for the development of PGP [64]. The accumulated evidence advocates in favour of a multifactorial condition during pregnancy and postpartum.

### 3.3. Diagnosis

#### 3.3.1. Joint Hypermobility

**Figure 2:**
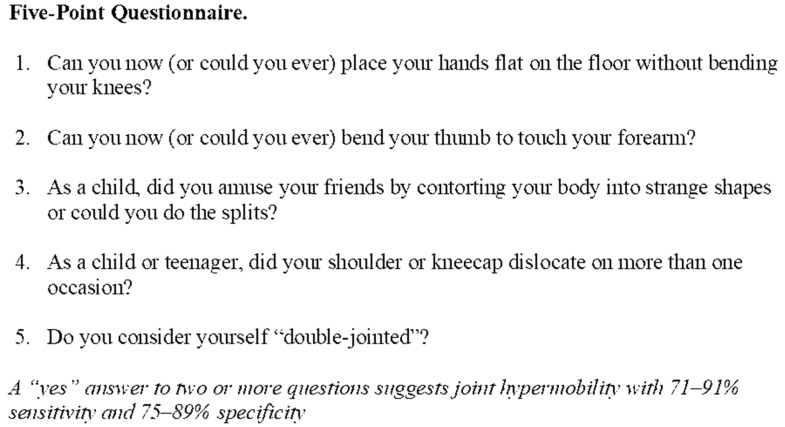
Five-point questionnaire. Adapted from Hakim and Grahame, 2003 [65,66,67].

Joint hypermobility can be diagnosed using either the Beighton score (Figure 1), or the Five-Point Questionnaire (5-PQ) (Figure 2) [14,65]. The Beighton score can be used when conducting a clinical examination and consists of five movements, four of which require bilateral assessment. This enables a total score to be calculated out of a maximum of nine. The 5-PQ on the other hand is a patient reported questionnaire consisting of five questions as its name suggests. It has the advantage of screening patients in circumstances where the Beighton score cannot be used, where patients have acquired hypomobility secondary to joint fusion or amputations for example [65].

#### 3.3.2. Hypermobility Spectrum Disorder

As discussed by Castori et al. HSD diagnoses are intended as alternative labels to describe symptomatic hypermobile joints based on their distribution, in individuals who do not meet the criteria for EDS, hEDS, or other causes of hypermobile joints such as neurological disorders, myopathies, chondrodysplasia and other connective tissue disorders. Findings for each subtype are summarised in Table 4. Musculoskeletal manifestations that may be present in such patients include micro- and macro-trauma, degenerative bone and joint disease, altered proprioception, muscle weakness and other physical traits [15].

#### 3.3.3. Hypermobility-Type Ehlers-Danlos Syndrome

The precise criteria for a diagnosis of hEDS is well described in the 2017 international classification and developed around three criteria which have been summarised below [16]:

1. Generalised joint hypermobility
  - Diagnosed by the Beighton score (Figure 1), with cut-off score for diagnosis varying by age
    - ≥6 for pre-pubertal children and adolescents
    - ≥5 for pubertal men and women up to the age of 50 years
    - ≥4 for those >50 years of age
  - If the Beighton score cannot be used, due to acquired hypomobility due to surgery and amputations for example, the Five-Point Questionnaire (5-PQ) (Figure 2) is to be used [65]
2. Associated features—divided into a further three subgroups A, B and C, where two or more of these subgroups are positive
  - Systemic manifestations of generalised connective tissue disorder
  - Positive family history, with one or more first degree relatives with hEDS
  - Musculoskeletal complications associated with hypermobile joints, including pain, instability and dislocations
3. Exclusion of other subtypes of EDS and other soft tissue disorders.

#### 3.3.4. Pelvic Girdle Pain

Diagnosis of PGP can be difficult and is guided mainly using tests conducted to provoke and elicit the pain. The SIJ and posterior elements of the pelvic girdle can be examined using the P4 posterior pelvic pain provocation test, Patrick’s FABER (flexion, abduction, external rotation of the hip) test, active straight leg raise, palpation of the long dorsal sacroiliac ligament and Gaenslen’s test [2,68,69,70,71]. The anterior pelvic girdle (pubic symphysis) can be examined with deep palpation of the pubic symphysis and the modified Trendelenburg’s test [68]. A variety of tests have been listed due to each test having high specificity, but poor sensitivity. By using an arsenal of examinations, the aim is to minimise false-negative results [2].

**Figure 3:**
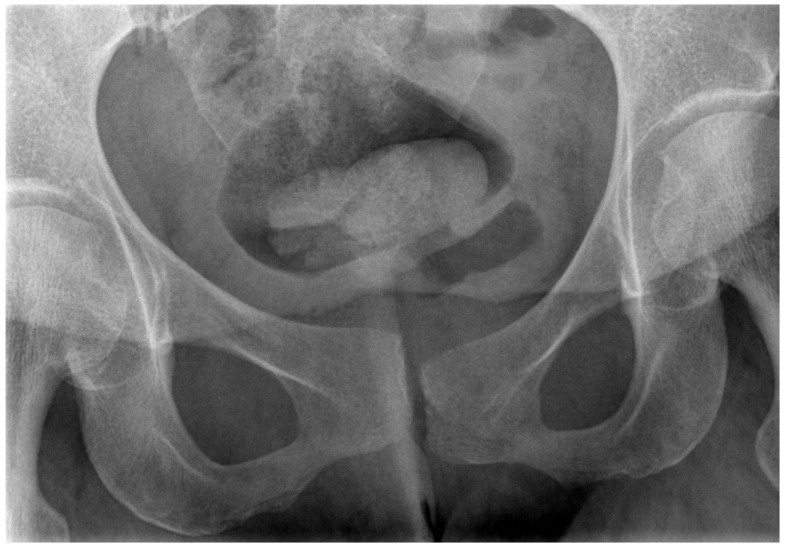
Pelvis flamingo view of a 36-year-old female suffering from hypermobility syndrome demonstrating abnormal movement of the pubic symphysis.

**Figure 4:**
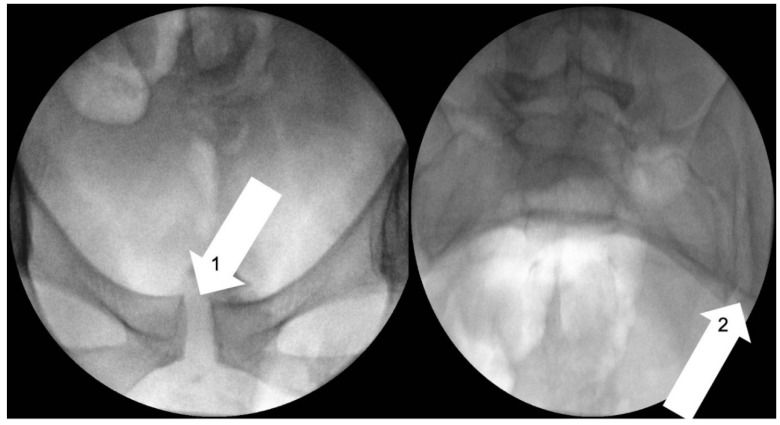
Fluoroscopic stress pelvic views radiograph in a 30-year-old female suffering from hypermobility syndrome demonstrating widening of pubic symphysis (Arrow 1) and left sacroiliac joint (Arrow 2).

**Figure 5:**
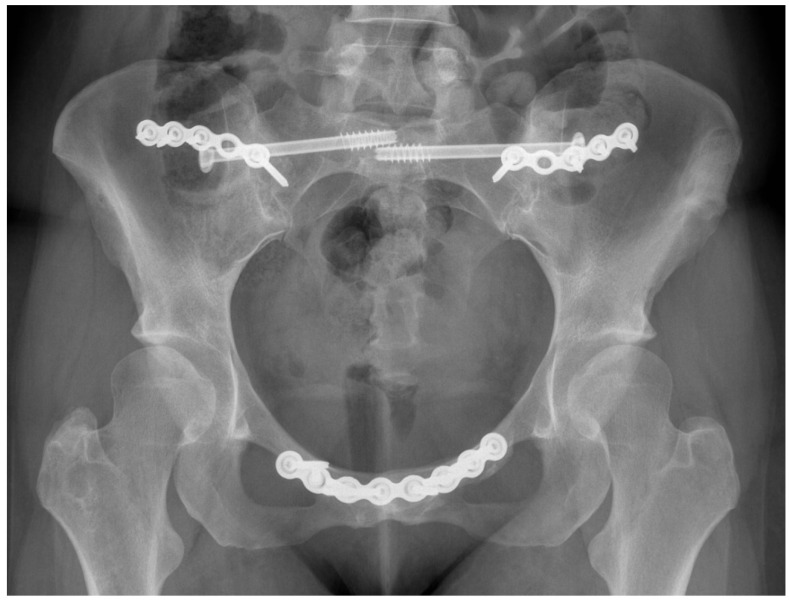
Anteroposterior pelvic radiograph of a 30-year-old female diagnosed with Ehlers-Danlos Syndrome demonstrating fusion of pubic symphysis and both sacroiliac joints (anterior plating, bone grafting and sacroiliac screw insertion).

**Figure 6:**
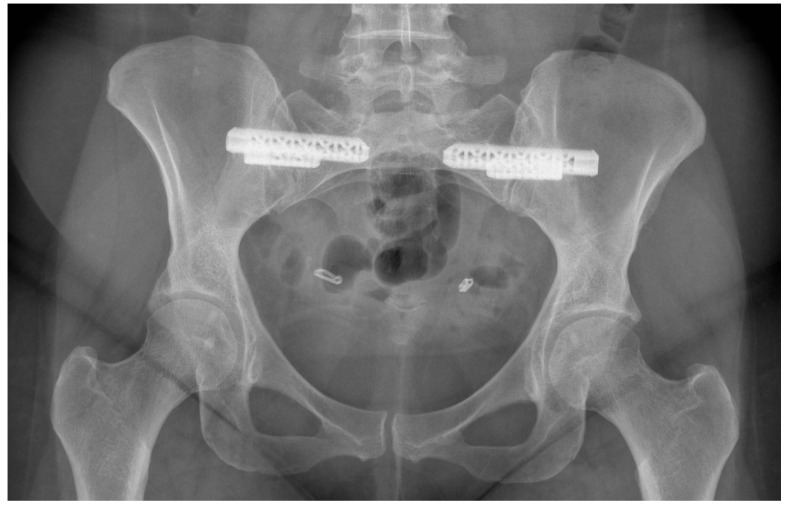
Anteroposterior pelvic radiograph in a 38-year-old female with hypermobility syndrome suffering from chronic sacroiliac joint pain who underwent stabilisation of bilateral sacroiliac joints with the iFuse implants.

The use of radiography and computerised tomography (CT) have not been promoted by the European guidelines for PGP as they provide little information on early degenerative disease. If magnetic resonance imaging (MRI) is available, it allows superior visualisation of early joint degeneration and inflammatory changes in the bones and surrounding soft tissues, as well as reducing exposure to radiation [68,72]. Radiographs may however be beneficial in determining pelvic instability with anteroposterior pelvic views with additional implementation of alternating single-leg stance (flamingo view) images, which may show significant pubis translation [58]. Please refer to Figure 3, Figure 4, Figure 5 and Figure 6 for examples of pre- and postoperative imaging.

### 3.4. Treatment

#### 3.4.1. Analgesia

Treatment of pelvic girdle pain (PGP) requires a holistic approach to address the many symptoms a patient may experience, but also the underlying aetiology. Pain control is of utmost importance to ensure comfort and continuation of function for the patient. This can be addressed using the analgesic ladder as well as the incorporation of neuropathic analgesia if indicated by signs of nerve irritation [73].

#### 3.4.2. Mental Health

It is also important to consider the impact of PGP on one’s mental health. Several studies have shown that females with chronic pelvic pain are at a significantly increased risk of suffering from anxiety and depression, even when compared to pain-free controls or population norms [74,75,76,77]. This is a significant factor to consider, as its implications extend beyond just the individuals mental health, which is an enormous challenge itself, where the lack of confidence as a consequence can limit their activities of daily living and quality of life.

Furthermore, it has been hypothesised that pain-related fear can lead patients into a vicious cycle where pain and injuries can persist because of emotional and behavioural responses to this pain [78]. It has also been found that the severity of pain correlates to fear avoidance beliefs, indicating that severe PGP could prove more challenging for rehabilitation and management [79]. With these additional challenges, integration of clinical psychology and psychiatry may play a vital role in pain management and rehabilitation for suffers of PGP, particularly in the postpartum population where this is additional concern for the care, safety and support required to manage a new-born [80,81]. A cohort study screened patients three months postpartum using the Edinburgh Postnatal Depression Scale and found that women with PGP were at 3.58 times the odds of screening positive for depression compared to women with no pelvic or lumbar back pain (*p* = 0.008) [82].

#### 3.4.3. Hypermobility Spectrum Disorder and Hypermobility-Type Ehlers-Danlos Syndrome Specialist Input

The management pathway of PGP first requires assessment to identify the possible underlying aetiology. This is crucial especially if a diagnosis of EDS is suspected, where a formal assessment by a Rheumatologist or specialist centre would be advocated to investigate systemic manifestations of the condition [83]. This is a vital step to identify any other potential concerning features an individual may possess that require further management (Table 5). This will also ensure that the individual receives the adequate support they require to improve their functional status and quality of life [83,84,85].

#### 3.4.4. Non-Surgical Interventions

Individuals with postpartum-PGP should be managed initially with a non-operative approach. A prospective cohort study investigating 130 women with PGP during and after pregnancy reported good prognosis from postpartum-PGP with 83% of individuals reporting substantial recovery within 6 weeks of delivery, 44% of whom recovered within 2 weeks [87]. The 2008 European guidelines for the diagnosis and treatment of pelvic girdle pain also advise conservative, non-invasive physiotherapy as first-line therapy with individualised exercise regimes designed to stabilise the pelvis and supporting structures [68].

In those with non-pregnancy related aetiology, physiotherapy is also the first step. As previously discussed, much of the pain is due to altered biomechanics throughout the pelvis. Physiotherapy attempts to restore the balance of the pelvis by manipulation of the soft tissues, whether it involves strengthening, stretching, improving flexibility, posture development, or core stabilisation to improve the biomechanics of one’s pelvic girdle [88,89]. This is also advised by Castori et al. for individuals with HSD/hEDS, with focus on stabilisation, manipulation, and postural hygiene [34]. Additional tools may also be used, such as pelvic belts and crutches to facilitate the rehabilitation process [35]. As proprioception can be compromised in individuals with JH, a particular emphasis should also be placed on developing this throughout the rehabilitation process [90].

If pelvic pain is persistent and not responsive to oral analgesia or conservative management, a more invasive approach may be adopted. Injection with a long-acting corticosteroid to the concerned joint(s) can be used [88,91,92]. Not only can this be effective from an analgesia perspective, it can be used as a diagnostic tool to assist in decision making when considering further management options, such as surgery, as done in previous studies [93,94,95].

#### 3.4.5. Surgical Interventions

The 2008 European pelvic girdle pain guidelines make no recommendations for surgery, but discuss the role of fusion surgery as a last resort in patients with resistant PGP, of pregnancy or traumatic aetiology, after poor response to other therapies [68]. A cross-sectional study conducted by Kibsgård et al. investigated the outcomes of 50 pelvic fusion patients using patient reported outcome measures (PROMs) [96]. A total of 21 patients underwent unilateral SIJ fusion, 25 patients had a bilateral SIJ fusion and 4 patients received fusion of both SIJs and the pubic symphysis. It also compared the outcomes of patients who underwent SIJ fusion against those that were managed without surgery, with an average follow-up period of 23 years for the surgical group, and 17 years for the non-surgical group.

It was concluded that 1-year post-surgery outcomes were maintained 23 years after surgery with good reported outcome by the patients, however this was not statistically different to those managed without surgery. This study is beneficial for the insight of joint fusion in PGP; however, it is limited by its lack of randomisation, making it prone to selection bias at the time of deciding the management option. In addition to this, the group sizes varied significantly with 50 individuals in the surgical group, and only 28 in the non-surgical. The symptomology of patients in each intervention arm were not elaborated on. However, information broadly assessing functional status by enquiring about impact on occupation and use of mobility aids showed no significant differences between the two groups, *p* = 0.32 and *p* = 0.21 respectively. Although PROMs were used for the long-term follow-up, the initial short-term outcome was analysed using CT scans conducted 1-year post-surgery with a vague assessment of its impact on quality of life by grading impact on the participants work. It would have been best if the PROMs were standardised and used for both short and long-term outcomes.

A further study conducted by Kibsgård et al. used a prospective design and standardised PROMs for assessment pre- and post-surgery in individuals with severe PGP [97]. This study found positive outcomes of SIJ fusion with significant reductions in the Oswestry Disability Index (ODI) scores and Visual Analogue Scale (VAS) pain scores, and also improvements in the Short Form-36 (SF-36) health survey. This study however is limited with a small sample size of 8 patients and concerning post-operative complications with an anterior approach to SIJ fusion. Three major complications included infection, complex regional pain syndrome with associated drop-foot, and loss of bladder sensation. Three patients also experienced transient loss of sensation in the region of the lateral femoral cutaneous nerve. The complications associated with the anterior approach could be minimised by either using a posterior approach or the iFuse minimally invasive surgery technique for SIJ fusion [98,99]. The latter method has been extensively reviewed by the National Institute for Health and Care Excellence (NICE) in the UK, and concluded that the evidence suggests improved pain, ODI and quality of life outcomes for the management of chronic SIJ pain, when compared to non-surgical therapy [99]. The above highlights the difficulty and severity of surgery as a method of managing pelvic girdle pain, justifying its role as an end-stage technique in its management. However, with recent developments in surgical instrumentation and techniques, it is becoming a more viable, and justifiable option for those with chronic and severe PGP.

Fusion of the pelvic joints is indeed a significant process with postoperative care that can be limiting to patients. Full weight bearing is to be avoided for at least 8 weeks to allow the fusion process to occur [93,97]. This could have a significant socioeconomic impact on a patient. Other risks associated with the reduced mobility include venous thromboembolism (although such a risk is minimised by administering low molecular weight heparin), non-union, morbidity at bone graft harvest site, and potential need to remove internal fixation plates at a later stage [100,101].

There are two broad categories of SIJ fusion, open and minimally invasive techniques. The open approach to the SIJ was first described in 1921 by Smith-Petersen which mentions a posterior transiliac approach to the SIJ [102]. Anterior approaches to the SIJ have also been described [97,103]. Smith et al. conducted a retrospective multi-centre study to compare the outcomes of open SIJ fusion compared minimally invasive techniques. They describe a posterior approach to the SIJ, with utilisation of an autologous bone graft from the posterior iliac crest and bone morphogenetic protein to facilitate fusion of the SIJ by packing into cages, followed by subsequent insertion of cancellous iliosacral lag screws [104]. This open technique was compared to the minimally invasive technique using the iFuse implant. This implant uses titanium, triangular-shaped devices that are coated in porous titanium plasma spray which allows biological fixation [104,105]. This study reports significantly lower operating time, estimated blood loss and length of admission when comparing the minimally invasive to open technique, all with *p* values < 0.001. It also reports a lower revision surgery rate for the minimally invasive technique, 3.5%, compared to 44% revision rate for open fusion cases [104]. The findings from this study should be taken with caution due to the lack of randomisation, significant difference in the mean age between groups and competing interests due to some authors having relationships with the producers of the implant, all which could introduce bias.

Notably, several minimally invasive implants which utilise either dorsal or lateral approaches in surgery are available, including triangular titanium implants, hydroxy-appetite coated screws, Simmetry and hollow modular anchorage screws. Martin et al. conducted a meta-analysis by assessing the impact of minimally invasive surgery for SIJ fusion on VAS and ODI, and found that both PROMs had significantly reduced (*p* < 0.05 for VAS and ODI) implying a significant improvement in pain as well functional outcomes of patients undergoing these procedures. Statistical analysis was not conducted against open SIJ fusion, nor between the different options for minimally invasive surgery [106]. A randomised controlled trial conducted by Dengler et al. compared the iFuse minimally invasive technique against conservative management for pain secondary to SIJ dysfunction, and found that those managed surgically had significantly improved VAS (*p* < 0.001), ODI (*p* < 0.001) and EuroQol 5-Dimension (EQ-5D) (*p* < 0.001) 24 months post-procedure [107].

This shows the benefit of minimally invasive techniques that can be provided to patients receiving SIJ fusion. A questionnaire to Spinal Surgeons enquiring about their experiences and preferences of minimally invasive and open found that minimally invasive techniques were the preference, with 80% of 121 surgeons reporting that they would not perform an open SIJ fusion procedure if it was the only option available [108].

Access to the pubic symphysis is typically obtained by an anterior approach using a Pfannenstiel incision for the purpose of fusion [97,100,109]. Once access to the pubic symphysis is obtained through dissection, a portion of the pubic symphysis joint is removed and repacked using an autologous graft from the iliac crest and a Matta plate to reconstruct the joint [97,100].

**Figure 7:**
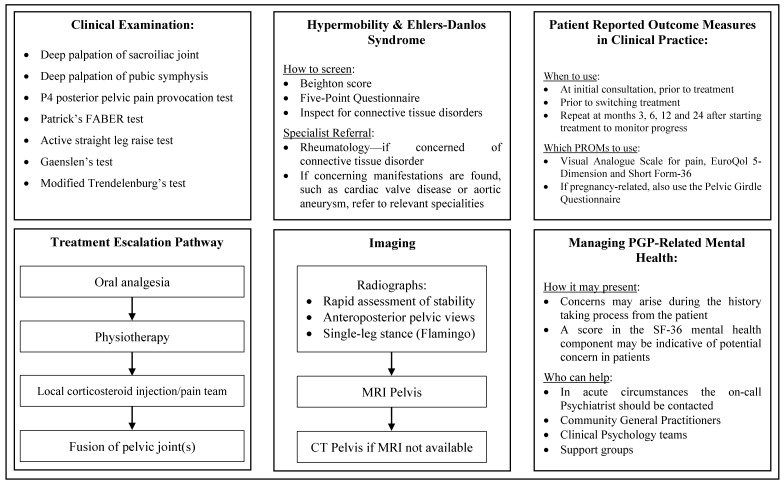
Summarised recommendations for the diagnosis and management of pelvic girdle pain (PGP) and hypermobility.

Previous studies have investigated the role of bone morphogenetic proteins in joint fusion, and have found them to be a safe and effective method of facilitating joint arthrodesis, by taking advantage of its ability to activate osteogenic pathways [93,110,111]. Giannoudis et al. in particular discussed its use in the pelvic girdle by using a combination of recombinant human bone morphogenetic protein-7 and purified type I collagen either on its own to fill the bone defects, or in conjunction with autologous bone grafts if the defect extended beyond two-centimetres in size [93]. This case series consisting of nine patients found no local or systemic complications associated with the bone morphogenetic protein-7, and bone healing was present in 89% of cases, with clinical and radiological fusion seen in a median average of 5 months [93]. In addition to bone morphogenetic protein, there is mention of mesenchymal stem cells and platelet-rich plasma potentially having a role in managing pelvic pain, particularly of the SIJ, however the lack of high-level evidence is currently limiting recommendation of this technique until further research is conducted [112]. Please refer to Figure 7 for a summary of recommendations for the diagnosis and management of patients with PGP and hypermobility.

## 4. Discussion

PGP is a complex disorder that is difficult to diagnose when considering its overlap in presentation with pathology of the pelvic viscera and spinal pathologies, making it primarily a diagnosis by exclusion. Assessment of existing literature is further challenged by the variety of names used to describe this condition, with similar difficulties associated with HSD and hEDS. Modern literature has however become more standardised by using PGP and HSD since the creation of newer classifications and guidelines [16,68]. This proved challenging when conducting a literature search for this review, where many papers were excluded due to being irrelevant. The broad search implemented was necessary, as when searching with the correct terms as discussed above, no results were found. It is for this reason that a broader search was conducted, with a screening process to filter the many publications relating to other causes of pelvic pain, which were not musculoskeletal.

Regardless, this review has identified HSD as a potential risk factor for the development of PGP, requiring a multidisciplinary approach to manage, provide adequate support, and appropriately diagnose rare conditions such as EDS. If not already incorporated into current clinical care pathways, a suggestion would be made to involve a rheumatologist if there is suspicion of HSD/hEDS, in order to ensure a holistic approach is pursued to identify any associated connective tissue problems, particularly those that are life-threatening such as aortic dilatations. Increasing awareness of patients and clinicians is also vital to ensure appropriate labelling of diagnoses related to PGP or HSD, as there is concern of incorrect or underdiagnosis of disease, with unknown exact prevalence [24].

A further challenge we face when interpreting current literature on management of outcomes is related to the broad variety of PROMs used in studies, particularly global health measures such as the SF-36, ODI, EQ-5D and Short Musculoskeletal Function Assessment which are not disease-specific as well as limiting translation and comparison of study findings, particularly where identical questionnaires were reported on differently [113,114]. PROMs related to the pelvis exist, such as the Pelvic Girdle Questionnaire, Majeed, Iowa and Orlando scores [115,116,117,118]. The latter three have been reviewed with concerns highlighted lack of emotional and mental health assessment and limited by floor and ceiling effects where individuals with impaired function may score well and vice versa. Moreover, they have unknown reliability and responsiveness particularly in longitudinal studies [119,120]. The Pelvic Girdle Questionnaire has been widely validated for use in pelvic pain in pregnant and postpartum individuals, however its use has not been validated in patients with PGP secondary to trauma and other causes [115,121,122,123,124,125,126,127]. The Pelvic Girdle Questionnaire does not contain any items that are gender or pregnancy specific. A future study exploring its validity as a functional outcome measure in trauma, pubic symphysis and SIJ dysfunction patients may highlight it as a suitable tool for such patients after its success in pregnant and postpartum women, potentially preventing the need to develop a new measure.

Several recommendations can be made based on the findings of this literature review. Upon diagnosis of PGP, completion of appropriate PROMs would be advised to monitor global health, functional status, and mental health using validated measures. This allows further assessment as to whether patients with impaired mental health would benefit from a psychological or psychiatric referral to improve their symptoms and rehabilitation. In addition, it would allow monitoring of functional status which can be repeated throughout treatment and allow more meaningful assessment of post-intervention outcomes. For pregnancy-related PGP, we would advise the use of the Pelvic Girdle Questionnaire as it is well-validated as previously discussed. For other causes of PGP we advise the use of validated global measures such as the VAS, EQ-5D and SF-36 or ODI, which can be compared to population norms, as the application of pelvic-specific outcomes for this population require further assessment [114,128,129].

It would also be advised to screen patients for JH in a formal manner using the Beighton score, or Five-Point Questionnaire if the Beighton score cannot be applied in clinic, or due to contraindications described earlier in the ‘*Diagnosis*’ section. Patients should be screened for further features of EDS to assist in the decision to make a referral to a Rheumatologist or EDS specialist, so patients have access to appropriate support services.

The findings included in this study have several limitations with regard to generalisation and of their approach toward JH. Self-diagnosis of JH would be deemed insufficient and question the validity of findings; diagnosis by a trained physician would be best. Moving forward, to improve the quality of available research, utilisation of the correct terminology and sufficient clinical examination is required to diagnose patients with PGP, HSD and EDS. To further develop our understanding of management options in this population, a multi-centre randomised control trial comparing conservative against surgical management is required utilising the latest available technologies and methods with the incorporation of validated disease-specific PROMs [99]. A multi-centre approach would also allow a large sample size to be generated as much of the current literature only includes small populations when assessing the outcomes of pelvic fusion surgery for those suffering from PGP.

## 5. Conclusions

In summary, ongoing literature would benefit from authors using the most recent and appropriate terminology and classifications for the diagnosis of PGP, HSD and hEDS. Patients presenting with PGP should formally be assessed for hypermobile joints, with support from connective tissue specialist colleagues for a formal diagnosis of HSD or hEDS. Standardised PROMs should be used in clinical practice and future research to facilitate translation and comparison of findings when monitoring post-intervention patient outcomes. Combining these points with a large randomised control trial will allow us to improve our understanding of the management of PGP in patients with a diagnosis of HSD and hEDS.

## Figures and Tables

**Table 1:** Spectrum of joint hypermobility phenotypes. Adapted from Castori et al., 2017 [15].

Asymptomatic Generalised Joint Hypermobility (Asymptomatic GJH)
Asymptomatic Peripheral Joint Hypermobility (Asymptomatic PJH)
Asymptomatic Localised Joint Hypermobility (Asymptomatic LJH)
Generalised Hypermobility Spectrum Disorder (G-HSD)
Peripheral Hypermobility Spectrum Disorder (P-HSD)
Localised Hypermobility Spectrum Disorder (L-HSD)
Historical Hypermobility Spectrum Disorder (H-HSD)
Hypermobility-Type Ehlers-Danlos Syndrome (hEDS)

**Table 2:** Summary of the five most common Ehlers-Danlos Syndrome (EDS) subtypes. Adapted from Malfait et al., 2017 [16].

EDS Subtype	Inheritance Pattern	Genetic Basis	Protein Affected	Common Clinical Signs (Not Extensive)
Classical EDS	Autosomal dominant	COL5A1	Type V collagen	Skin: hyperextensible, fragile, soft, atrophic scarring
		COL1A1	Type I collagen	Other: generalised-JH, hernias
Classical-like EDS	Autosomal recessive	TNXB	Tenascin XB	Skin: hyperextensible, velvety, absence of atrophic scarring, easy bruising skin
				Other: generalised-JH, foot deformities, oedema, pelvic organ prolapse
Cardiac-Valvular EDS	Autosomal recessive	COL1A2	Type I collagen	Progressive cardiac valve disease
				Skin: hyperextensible, atrophic scars, easy bruising
				Other: Joint hypermobility, hernia, foot deformity
Vascular EDS	Autosomal dominant	COL3A1	Type III collagen	Arterial rupture at young age, spontaneous sigmoid colon perforation, uterine rupture, peripartum perineal tears, carotid-cavernous sinus fistula, thin skin, varicose vein
		COL1A1	Type I collagen	
Hypermobile EDS	Autosomal dominant	Unknown	Unknown	Generalised-JH, absence of skin fragility, mild skin hyperextensibility, unexplained striae, pelvic organ prolapse, dental crowding, hernias

**Table 3:** Summary of included articles from literature search.

Article Title	Publication Year	First Author	Journal	Article Type	Relevance
Hypermobility and peripartum pelvic pain syndrome in pregnant South African women [31]	1999	Van Dongen, PW	International Journal of Gynecology & Obstetrics	Cross-Sectional Study	This study included 509 South African pregnant women, of which only 4.9% suffered from hypermobility (Beighton score ≥5/9). No significant correlation was determined between peripartum pelvic pain and hypermobility. Only 20 cases of peripartum pelvic pain were recorded in the entire study population. There is no mention of JH prevalence in these 20 patients with pelvic pain.
Low back pain and pelvic pain during pregnancy: prevalence and risk factors [32]	2005	Mogren, IM	Spine	Cross-Sectional Study	Identified that women with diagnosed hypermobility were at 1.79 times the odds (95% confidence interval 1.14–2.80) of developing low back and pelvic pain during pregnancy compared to those with normal joints (*p* = 0.012). Did not differentiate between low back pain and pelvic pain.
Body mass index (BMI), pain and hyper-mobility are determinants of long-term outcome for women with low back pain and pelvic pain during pregnancy [33]	2006	Mogren, IM	European Spine Journal	Cross-Sectional Study	Identified that women with diagnosed hypermobility were not at increased risk of low back and pelvic pain 6-months postpartum (*p* = 0.123). Grouping of women with diagnosed hypermobility and whose who *perceived* themselves to be hypermobile found statistical significance in increased risk of low back and pelvic pain 6-month postpartum (*p* = 0.042), odds ratio 1.56 (95% confidence interval 1.01–2.40).
Management of pain and fatigue in the joint hypermobility syndrome (a.k.a. Ehlers-Danlos syndrome, hypermobility type): principles and proposal for a multidisciplinary approach [34]	2012	Castori, M	American Journal of Medical Genetics Part A	Literature Review	Mentions pelvic ring instability are likely more common in hypermobility spectrum disorder (HSD). Also states trunk stabilisation as treatment but is usually ineffective due to high risk of instability recurrence.
Ehlers-Danlos Syndrome-Hypermobility Type: A Much Neglected Multisystemic Disorder [35]	2016	Gazit, Y	Rambam Maimonides Medical Journal	Literature Review	Discusses increase in joint laxity and pain during pregnancy. Pelvic pain management is discussed in the form of pelvic belts, crutches and bed rest. Mentions women with Ehlers-Danlos Syndrome, mainly hypermobility subtype, are more likely to suffer from pelvic pain and instability (26%) compared to those without the condition (7%), *p* < 0.05 [29].
Joint Hypermobility among Female Patients Presenting with Chronic Myofascial Pelvic Pain [39]	2019	Hastings, J	PM&R	Retrospective Case-Control Study	This study included 318 women who were diagnosed with chronic myofascial pelvic pain during a 1-year period. Prevalence of generalised-HSD was 24% in this sample. Secondary outcomes found women with generalised-HSD were at 7.46 times the odds of low back pain compared to those with normal joints (*p* = 0.02).
Sacroiliac Joint Hypermobility Biomechanics and What it Means for Health Care Providers and Patients [36]	2019	Enix, DE	PM&R	Literature Review	Discusses biomechanical causes of pelvic pain, and approaches to physiotherapy treatment. Advise that manual manipulation therapy may be useful as an initial treatment, but not suitable long-term. A care plan including active and passive strategies that engage patients are likely to improve compliance and outcomes.
Biomechanics of the Sacroiliac Joint: Anatomy, Function, Biomechanics, Sexual Dimorphism, and Causes of Pain [37]	2020	Kiapour, A	International Journal of Spine Surgery	Literature Review	States hypermobility is a potential cause for pain at the sacroiliac joint (SIJ), mentioning that female SIJs have greater mobility when compared to males, resulting in greater stresses, loads and pelvic ligament strains [40].
Sacroiliac Joint Injury [38]	2020	Dydyk, AM	StatPearls	Book	States fusion surgery or pregnancy can lead to either hypermobility or hypomobility of the SIJ, which can cause pain. Also highlights several causes of SIJ injury such as trauma, leg-length discrepancy, obesity, pregnancy, and hypermobility.
The Association of Self-Reported Generalized Joint Hypermobility with pelvic girdle pain during pregnancy: a retrospective cohort study [28]	2020	Ahlqvist, K	BMC Musculoskeletal Disorders	Cohort Study	A 5-point questionnaire was used to determine self-reported generalised joint hypermobility (GJH). 2455 women from 144 antenatal clinics in Sweden were included. Prevalence of overall self-reported GJH was 28.7%. Women with GJH had higher odds of developing pelvic girdle pain during pregnancy than those without GJH, *p* = 0.001 (odds-ratio 1.27, 95% confidence interval 1.11–1.47).

**Table 4:** Summary of hypermobility spectrum disorder subtypes. Adapted from Castori et al., 2017 [15].

Hypermobility Spectrum Disorder (HSD) Subtype	Beighton Score	Notes
Generalised-HSD	Positive	High suspicion of potential hypermobility-type Ehlers-Danlos Syndrome (hEDS), requiring thorough assessment. Mainly consists of individuals with generalised joint hypermobility (GJH) and secondary musculoskeletal manifestations, but do not meet full criteria for hEDS.
Peripheral-HSD	Usually negative	Joint hypermobility (JH) limited to hands and feet with one or more secondary musculoskeletal manifestations.
Localised-HSD	Negative	JH at single or small group of joints with secondary musculoskeletal manifestations.
Historical-HSD	Negative	Self-reported GJH using the five-point questionnaire, with a negative Beighton score, plus musculoskeletal manifestations.

**Table 5:** Possible systemic features of hypermobility-type Ehlers-Danlos Syndrome and hypermobility spectrum disorders. Adapted from Castori, 2012 [86].

Dermatological	Skin hyperextensibility—to a lesser extent than seen in Classical Ehlers-Danlos Syndrome
	Velvety, soft skin texture
	Absence of skin fragility
	Striae formation
	Herniation of bowel or muscle
	Capillary fragility and wound healing defects such as atrophic scars
Orthopaedic	Congenital capsule-ligamentous laxity
	Joint hypermobility
	Joint instability
	Joint dislocations
	Soft tissue injuries
	Spine: hyperkyphosis, hyperlordosis, scoliosis
	Fixed subluxations of: costochondral, sternoclavicular, distal radioulnar, first carpometacarpal joints
	Cubitus valgus
	Genuum valgum
	Hallux valgus
	Femur anteversion
	Flexible flatfoot
Gynaecological	Irregular menses
	Menorrhagia
	Metorrhagia
	Dysmenorrhea
	Uterine prolapse
	Urinary stress incontinence
Neurological	Altered myopathic electrophysiology
	Reduced sensation
	Muscle weakness
	Myalgia and cramps
	Neuropathy
	Chronic/recurrent pain—musculoskeletal, neuropathic and visceral
	Sleep disturbance
	Migraines and headaches
	Impaired joint proprioception
Mucosal & Oral	Dryness of eyes, mouth and vagina
	Mucosal fragility causing spontaneous epistaxis and gingival bleeding
	Agenesis or absence of lingual frenulum
	Temporomandibular joint dysfunction
Cardiac	Cardiac valve disease
	Aortic root dilatation
	Autonomic cardiac dysfunction—orthostatic intolerance, postural tachycardia syndrome
Pulmonary	Asthmatic and atopic symptoms
	Increased lung volumes
	Impaired gas exchange
	Upper and lower airway collapse
Ocular	Blue sclera
	Blepharochalasis
	Antimongoloid palpebral slant
	Myopia
	Xerophthalmia
Gastrointestinal	Constipation (slow transit)/diarrhoea
	Gastroesophageal reflux
	Gastritis
	Hiatus hernia
	Crohn’s disease
	Abdominal pain
	Faecal incontinence
Psychiatric	Anxiety
	Depression
	Panic disorder
	Personality disorder

## Abbreviations

*5-PQ*	Five-Point Questionnaire
*BMI*	Body Mass Index
*CT*	Computerised Tomography
*EDS*	Ehlers-Danlos Syndrome
*EQ-5D*	EuroQol 5-Dimension
*G-HSD*	Generalised Hypermobility Spectrum Disorder
*GJH*	Generalised Joint Hypermobility
*hEDS*	Hypermobility-type Ehlers-Danlos Syndrome
*H-HSD*	Historical Hypermobility Spectrum Disorder
*HSD*	Hypermobility Spectrum Disorder
*JH*	Joint Hypermobility
*L-HSD*	Localised Hypermobility Spectrum Disorder
*LJH*	Localised Joint Hypermobility
*MRI*	Magnetic Resonance Imaging
*NICE*	National Institute for Health and Care Excellence
*ODI*	Oswestry Disability Index
*PGP*	Pelvic Girdle Pain
*P-HSD*	Peripheral Hypermobility Spectrum Disorder
*PJH*	Peripheral Joint Hypermobility
*PROMs*	Patient Reported Outcome Measures
*SF-36*	Short Form-36
*SIJ*	Sacroiliac Joint
*VAS*	Visual Analogue Scale
